# Vaccination and Syphilis

**Published:** 1865-04

**Authors:** 


VACCINATION AND SYPHILIS.—An important discussion is now pending at the Academy of Medicine of Paris. M. Depaul, one of the members, and reporter on the progress of vaccination, has incorporated in his report the late calamitous propagation of syphilis by vaccination. He expresses himself very strongly on the subject, and is likely to perplex very much the secretary of state, to whom the report is addressed. M. Ricord lately, in an excellent speech, combated the views of M. Depaul, and a lively debate is expected.

